# Comparison of gastric inflammation and metaplasia induced by *Helicobacter pylori* or *Helicobacter felis* colonization in mice

**DOI:** 10.1128/spectrum.00015-24

**Published:** 2024-04-29

**Authors:** Sara R. Druffner, Shrinidhi Venkateshwaraprabu, Stuti Khadka, Benjamin C. Duncan, Maeve T. Morris, Emel Sen-Kilic, Fredrick H. Damron, George W. Liechti, Jonathan T. Busada

**Affiliations:** 1 Department of Microbiology, Immunology and Cell Biology, West Virginia University School of Medicine, Morgantown, West Virginia, USA; 2 Department of Microbiology and Immunology, Uniformed Services University of the Health Sciences, Bethesda, Maryland, USA; Tainan Hospital, Ministry of Health and Welfare, Tainan, Taiwan

**Keywords:** *Helicobacter pylori*, *Helicobacter felis*, gastric cancer, CagA, stomach, inflammation

## Abstract

Gastric cancer is the fifth most diagnosed cancer in the world. Infection by the bacteria *Helicobacter pylori* (HP) is associated with approximately 75% of gastric cancer cases. HP infection induces chronic gastric inflammation, damaging the stomach and fostering carcinogenesis. Most mechanistic studies on gastric cancer initiation are performed in mice and utilize either mouse-adapted strains of HP or the natural mouse pathogen *Helicobacter felis* (HF). Here, we identified the differences in gastric inflammation, atrophy, and metaplasia associated with HP and HF infection in mice. PMSS1 HP strain or the CS1 HF strain were co-cultured with mouse peritoneal macrophages to assess their immunostimulatory effects. HP and HF induced similar cytokine production from cultured mouse peritoneal macrophages revealing that both bacteria exhibit similar immunostimulatory effects *in vitro*. Next, C57BL/6J mice were infected with HP or HF and were assessed 2 months post-infection. HP-infected mice caused modest inflammation within both the gastric corpus and antrum, and did not induce significant atrophy within the gastric corpus. In contrast, HF induced significant inflammation throughout the gastric corpus and antrum. Moreover, HF infection was associated with significant atrophy of the chief and parietal cell compartments and induced the expression of pyloric metaplasia (PM) markers. HP is poorly immunogenic compared to HF. HF induces dramatic CD4+ T cell activation, which is associated with increased gastric cancer risk in humans. Thus, HP studies in mice are better suited for studies on colonization, while HF is more strongly suited for studies on the effects of gastric inflammation on tumorigenesis. ** **

**IMPORTANCE:**

Mouse infection models with *Helicobacter* species are widely used to study *Helicobacter* pathogenesis and gastric cancer initiation. However, *Helicobacter pylori* is not a natural mouse pathogen, and mouse-adapted *H. pylori* strains are poorly immunogenic. In contrast, *Helicobacter felis* is a natural mouse pathogen that induces robust gastric inflammation and is often used in mice to investigate gastric cancer initiation. Although both bacterial strains are widely used, their disease pathogenesis in mice differs dramatically. However, few studies have directly compared the pathogenesis of these bacterial species in mice, and the contrasting features of these two models are not clearly defined. This study directly compares the gastric inflammation, atrophy, and metaplasia development triggered by the widely used PMSS1 *H. pylori* and CS1 *H*. felis strains in mice. It serves as a useful resource for researchers to select the experimental model best suited for their studies.

## INTRODUCTION

Gastric cancer is the fifth most common cancer and the fourth leading cause of cancer deaths worldwide (1). *Helicobacter pylori* (HP) infection is the primary risk factor for gastric cancer, contributing to 75% of the global gastric cancer burden (2). HP infection is extremely prevalent, infecting approximately 50% of the world’s population and 1%–3% of infected individuals will develop cancer (3). Stomach infection by HP induces a well-established histopathological cascade, driving chronic inflammation of the gastric mucosa, which in turn causes widespread epithelial damage and metaplasia, eventually resulting in adenocarcinoma (2, 4). Despite the strong link between HP and gastric cancer, chronic inflammation is both necessary and sufficient for gastric cancer initiation. A host of previous studies have shown that *Helicobacter-*independent chronic gastric inflammation induced by the overexpression of proinflammatory cytokines, surgical depletion of anti-inflammatory hormones, or through autoimmune gastritis induces gastric atrophy, metaplasia, and tumorigenesis (5–9). Similarly, polymorphisms resulting in higher levels of the proinflammatory cytokines IL1B or TNF and lower levels of the anti-inflammatory cytokine IL10 are associated with increases in HP-related gastric cancer (10, 11). In contrast, multiple immune-deficient mouse models are protected from the gastric atrophy associated with *Helicobacter* infection (12–14). Although it is well established that chronic inflammation modifies gastric cancer risk, the inflammatory phenotypes that promote cancer initiation remain poorly defined.

The vast majority of mechanistic studies are performed in mouse models. However, studies in mice pose specific challenges. HP is not a natural mouse pathogen, and clinical isolates typically require serial passage through mice to enhance mouse colonization (15, 16). Mouse-adapted HP strains, such as the commonly used SS1 strain, have lost the expression of several virulence factors that increase disease severity in humans, and they rarely cause severe disease in mice (17). CagA is the best-described example of loss of virulence factors during mouse adaptation. The CagA pathogenicity island is the best-known correlate of cancer risk. Encoding a type IV secretion system (T4SS) and CagA oncoprotein, this system injects CagA and other bacterial components into host epithelial cells, exacerbating inflammation and inducing host cell mutations (18). In the SS1 strain, CagA translocation to host epithelial cells is lost due to rearrangements in CagY (19). Although the pre-mouse-adapted SS1 strain (PMSS1) maintains CagA expression and T4SS function *in vitro*, CagA translocation is lost after 3–4 months of mouse colonization (20, 21).

*Helicobacter felis* (HF) is a close relative to HP that was originally isolated from the gastric mucosa of a cat (22). The HF genome shares more than 60% homology with HP and encodes many orthologs of HP essential genes that are required for gastric colonization (23). HF readily colonizes mice and quickly triggers massive gastric inflammation and atrophic gastritis within 2 months post-infection (24). Long-term infection over 12–24 months induces dysplasia and occasionally non-invasive adenocarcinoma (25). Although HF does colonize the human stomach, it is not a gastric cancer risk factor (26, 27). Moreover, HF does not express the CagA pathogenicity island, potentially limiting its ability to promote tumorigenesis in experimental models (23). However, HF’s robust induction of gastric inflammation makes it well suited for studies on how severe inflammation affects the gastric mucosa.

Both HP and HF are widely used to study gastric cancer initiation, but the strengths and weaknesses of each bacteria species may not be readily apparent. Typically, studies have elected to use one species or the other, and few have directly compared either of the bacteria to one another or their effects on the host (28, 29). The goal of this study was to directly compare the effects of HP and HF on immune-cell activation *in vitro* and their effects on gastric inflammation, atrophy, and metaplasia development in mice. We infected C57BL/6J mice with the HP PMSS1 strain, the most widely used CagA+ strain, and the HF CS1 strain. Mice were assessed 2 months post-infection to ensure that HP still maintained CagA function. Our findings indicate that both bacterial species exhibit a similar ability to stimulate macrophages *in vitro*, but that HF induces significantly more intense and widespread gastric inflammation and atrophy in mice. Although both bacterial strains have specific utility, this study aims to serve as a resource to help researchers choose the bacterial model that best suits their experimental goals.

## MATERIALS AND METHODS

### Animal care and treatment

All mouse studies were performed with approval by the West Virginia University Animal Care and Use Committee. C57BL/6J mice were purchased from the Jackson Laboratories. Mice were administered standard chow and water *ab libitum* and maintained in a temperature- and humidity-controlled room with standard 12-hour light/dark cycles. Eight-week-old mice were mock infected or infected with HP or HF by oral gavage. Mock mice received 500-µL sterile Brucella broth, and infected mice were inoculated with 500 µL of Brucella broth containing 10^9^ CFU of the respective bacteria two times 24 hours apart. For the HP infection studies, both male and female mice were used. While for the HF infection studies, only female mice were used as there are significant sex differences in the response to HF. For peritoneal macrophage isolation, mice received a single intraperitoneal (IP) injection of 1-mL sterile Brewers Thioglycollate media (Sigma-Aldrich). After 4 days, the peritoneal lavage was plated, and nonadherent cells were removed by washing with prewarmed 1x phosphate-buffered saline (PBS).

### Bacterial preparation

HF (ATCC 49179) was grown on tryptic soy agar plates (BD Biosciences) with 5% defibrinated sheep blood (Hemostat Labs) and 10 µg/mL vancomycin (Alfa Aesar) under microaerophilic conditions (5% O_2_ and 10% CO_2_) at 37°C for 2 days. HF was then harvested and transferred to Brucella broth (Research Products International) containing 5% fetal bovine serum (FBS) (R&D Systems) and 10 µg/mL vancomycin, and was grown overnight at 37°C under microaerophilic conditions with agitation. Bacteria were centrifuged and resuspended in fresh Brucella broth without antibiotics before spectrophotometry and mouse infection.

*H. pylori* PMSS1(30) (a gift from Manuel Amieva, Stanford University) was inoculated in Brucella broth containing 10% fetal bovine serum and 10 µg/mL vancomycin, and was grown overnight at 37°C under microaerophilic conditions with agitation. Bacteria were centrifuged and resuspended in fresh Brucella broth without antibiotics before spectrophotometry and mouse infection.

### Tissue preparation

Mice were euthanized 2 months post-inoculation. Stomachs were removed and opened along the greater curvature and were washed in phosphate-buffered saline to remove gastric contents. One side of the stomach was fixed overnight in 4% paraformaldehyde at 4°C and cut into strips. The strips were either cryopreserved in 30% sucrose and embedded in optimal cutting temperature (OCT) media or transferred into 70% ethanol and submitted to the West Virginia University histology core for routine processing, embedding, sectioning, and H&E staining. For histology, strips were taken from the middle of the gastric corpus, avoiding the lesser curvature. A 2-mm biopsy was removed from the other side of the corpus for RNA isolation and was immediately snap-frozen in liquid nitrogen. The remainder of the corpus was disassociated into a single cell suspension for flow cytometry as described below.

### Histology

Immunostaining was performed using standard methods. Briefly, 5-µm stomach cryosections were incubated with anti H+/K+ ATPase antibodies (clone 1H9, MBL Life Science), MIST1 (Cell Signaling Technologies), CD45 (clone 104; Biolegend), or CD44v9 (Cosmo Bio) for 1 hour at room temperature or overnight at 4°C. Sections were incubated in secondary antibodies for 1 hour at room temperature. Fluorescence-conjugated *Griffonia simplicifolia* lectin (GSII; Thermo Fisher Scientific) was added with secondary antibodies where indicated. Sections were mounted with Vectastain mounting media containing 4’,6-diamidino-2-phenylindole (DAPI) (Vector Laboratories). Images were obtained using a Zeiss 710 confocal laser-scanning microscope (Carl-Zeiss) and running Zen Black (Carl-Zeiss) imaging software.

### RNA isolation and qRT-PCR

For gastric tissue, RNA was extracted in TRIzol (Thermo Fisher Scientific) and precipitated from the aqueous phase using an equal volume of 100% ethanol. The mixture was transferred to an RNA isolation column (Omega Bio-Tek), and the remaining steps were followed according to the manufacturer’s recommendations. For peritoneal macrophages, RNA was isolated using the MicroElute Total RNA kit (Omega Bio-Tek). RNA was treated with RNase-free DNase I (Omega Bio-Tek) as part of the isolation procedure. Reverse transcription followed by quantitative PCR was performed in the same reaction using the Universal Probes One-Step PCR kit (Bio-Rad Laboratories) and the TaqMan primers/probe mixtures (all from Thermo Fisher Scientific): *Ppib* (Mm00478295_m1), *Wfdc2* (Mm00509434_m1), *Il13* (Mm00434204_m1), *Ifng* (Mm01168134_m1), *Cftr* (Mm00445197_m1), *Tnf* (Mm00443258_m1), and *Il1b* (Mm00434228_m1). Relative gene expression was normalized to *Ppib*.

### Flow cytometry

Corpus tissue from euthanized mice was washed in Hanks Balanced Salt Solution without Ca^2+^ or Mg^2+^ containing 5 mM HEPES, 5 mM EDTA, and 5% FBS at 37°C for 20 minutes. The tissue was then washed in Hanks Balanced Salt Solution with Ca^2+^ or Mg^2+^ briefly and then digested in 1 mg/mL collagenase (Worthington) for 30 minutes at 37°C. After digestion, the tissue fragments were pushed through a 100-µM strainer and then rinsed through a 40-µM strainer before debris was removed through an Optiprep (Serumwerk) density gradient. Fc receptors were blocked with TruStain (Biolegend) and then stained with antibodies for 20 minutes on ice. The following antibodies were used: CD45.2 (clone 104), CD3e (clone 145–2C11), B220 (clone RA3-6B2), CD4 (clone GK1.5), CD8a (clone 53–6.7), CD11b (clone M1/70), MHCII (clone M5/114.15.2), Ly6g (clone 1A8), F4/80 (clone BM8), and SiglecF (E50-2440). Actinomycin D (A1310, Invitrogen) was used to label dead cells. Cells were analyzed on a Cytek Aurora spectral flow cytometer (Cytek Biosciences). Flow cytometry analysis was performed using Cytobank (Beckman Coulter).

### Peritoneal macrophage treatment and cytokine array

Peritoneal macrophages were treated with vehicle (sterile Brucella broth) or a 1:2 ratio of macrophages to HP or HF. Cells were washed and collected 3 hours posttreatment for RNA isolation. Media was collected 24 hours posttreatment for cytokine analysis. Cytokines were assessed using the Mouse C3 cytokine array (RayBiotech) following the manufacturer’s protocol. The arrays were imaged with an iBright 1500 (Thermo Fisher Scientific), and dot density was measured using the on-board analysis software. The cytokines were considered expressed if their pixel density was 1.5 times higher than the negative control dots printed on the cytokine array. A heatmap was generated using the Morpheus heat map tool (Broad Institute).

### Statistical analysis

All error bars are ±SD of the mean. The sample size for each experiment is indicated in the figure legends. Experiments were repeated a minimum of two times. Statistical analyses were performed using a one-way analysis of variance with the post hoc Tukey *t*-test when comparing three or more groups, or by an unpaired *t*-test when comparing two groups. Statistical analysis was performed by GraphPad Prism 10 software. Statistical significance was set at *P* ≤ 0.05. Specific *P* values are listed in the figure legends.

## RESULTS

### *H. felis* and *H. pylori* exhibit similar immunostimulatory effects *in vitro*

**Fig 1:**
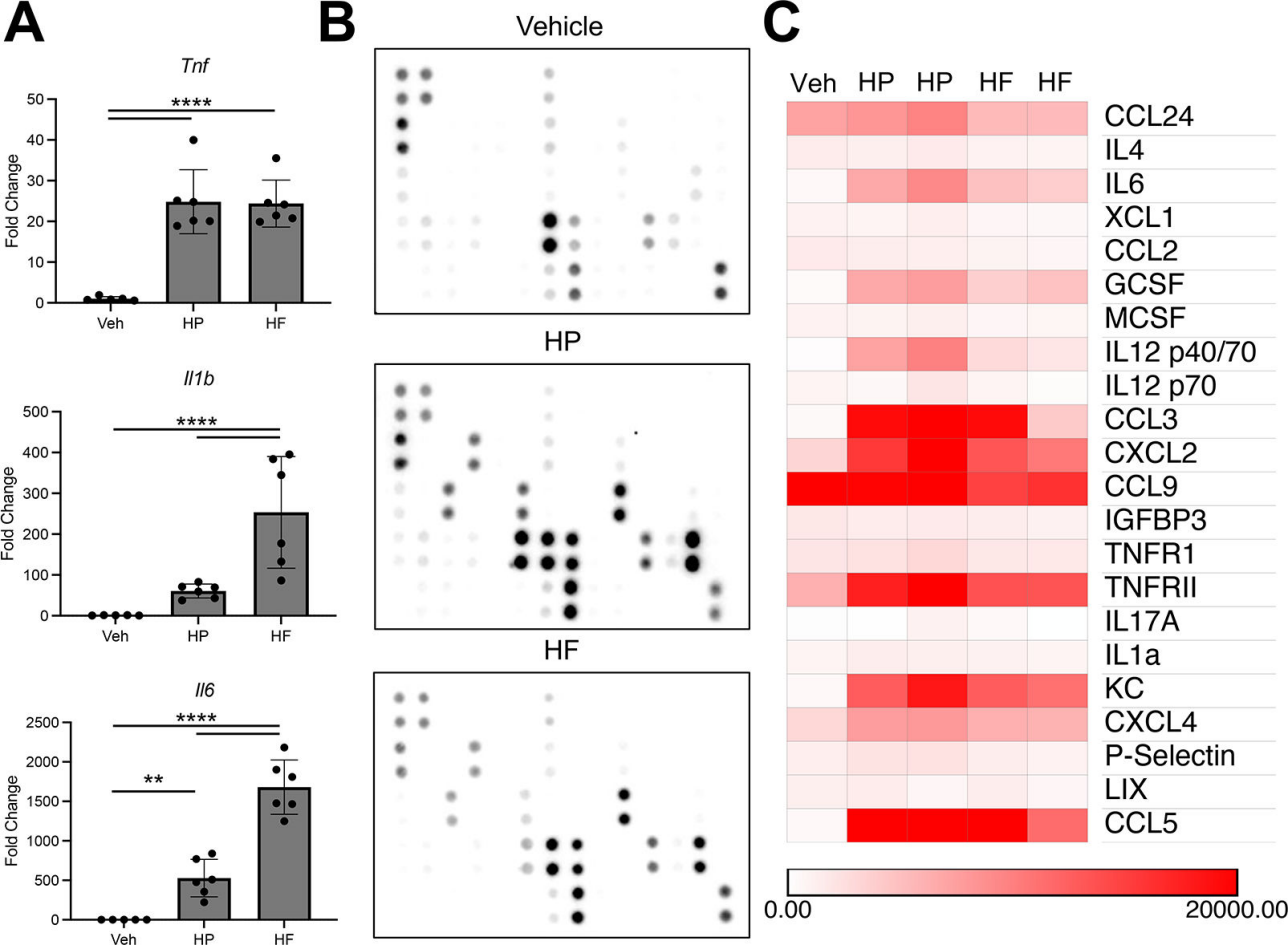
*H. pylori* and *H. felis* elicit similar activation of macrophages *in vitro*. (**A**) Quantitative RT-PCR of the indicated genes using RNA isolated from cultured peritoneal macrophages 3 hours after mock stimulation or stimulation with a 1:1 ratio of *H. pylori* or *H. felis*. *n* ≥ 5. **P ≤ 0.01 ****P ≤ 0.0001. (**B**) Representative dot plot cytokine arrays. Arrays were probed with cell culture media from peritoneal macrophages stimulated for 24 hours with a 1:1 ratio of *H. pylori* or *H. felis*. Dot labels are found in Table 1. (**C**) Heatmap of the cytokine arrays is shown in (**B**). Cytokines were considered expressed if the dot density was 1.5-fold above the background. *n* ≥ 3.

HP has evolved to avoid the host immune system (30). Mutations in HP lipopolysaccaride (LPS) and flagellar proteins avoid binding of TLR4 and TLR5, respectively (28, 31). The enhanced immunogenicity of HF *in vivo* raises the possibility that HF is intrinsically more immunostimulatory than HP. We isolated thioglycolate-induced peritoneal macrophages from C57Bl6/J mice to examine the immunostimulatory effects of HP and HF. The cell cultures were challenged with a 2:1 cell ratio of HP or HF for 3 hours before assessment of proinflammatory cytokine expression by qRT-PCR. Challenge with either bacterium induced similar levels of the *Tnf*, while *Il6* and *Il1b* induction was significantly higher in HF-challenged macrophages (Fig. 1A). Next, we used the same experimental system to measure bacterial induction of cytokines. Macrophages were co-cultured with either HF or HP for 24 hours, and the cell culture media was assessed by a mouse 62 cytokine dot plot array (Table 1). For this assay, a cytokine was considered detectable if the densitometry was 1.5-fold above the negative control background. Twenty-two cytokines were detected in the tissue culture media. Several cytokines were dramatically induced by bacterial co-culture, but surprisingly, there were no notable differences between the cytokine expression profiles of HP or HF-stimulated macrophages (Fig. 1B and C). These data demonstrate that HP and HF have a similar propensity to stimulate macrophages *in vitro*, suggesting that both strains are similarly immunogenic.

**TABLE 1:** Cytokine array dot plot key

	A	B	C	D	E	F	G	H	I	J	K	L	M	N
1	Pos	Pos	Neg	Neg	Blank	AXL	CXCL13	TNFSF8	TNFRSF8	TNFRSF5	CRG2	CCL27	CXCL16	CCL11
2														
3	CCL24	TNFSF6	CX3CL1	GCSF	GMCSF	IFNɣ	IGFBP3	IGFBP5	IGFBP6	IL1⍺	IL1β	IL2	IL3	IL3Rβ
4														
5	IL4	IL5	IL6	IL9	IL10	IL12 p40/p70	IL12 p70	IL13	IL17A	CXCL1	Leptin R	Leptin	LIX	CD62L
6														
7	XCL1	CCL2	MCP5	MCSF	CXCL9	CCL3	MIP1ɣ	MIP2	CCL19	CCL20	CXCL4	P-Selectin	CCL5	SCF
8														
9	SDF1	CCL17	CCL1	CCL25	TIMP1	TNF⍺	TNFRSF1A	TNFRSF1B	TPO	CD106	VEGFA	Blank	Blank	POS
10														

### *H. felis* infection induces severe inflammation of the gastric corpus

**Fig 2:**
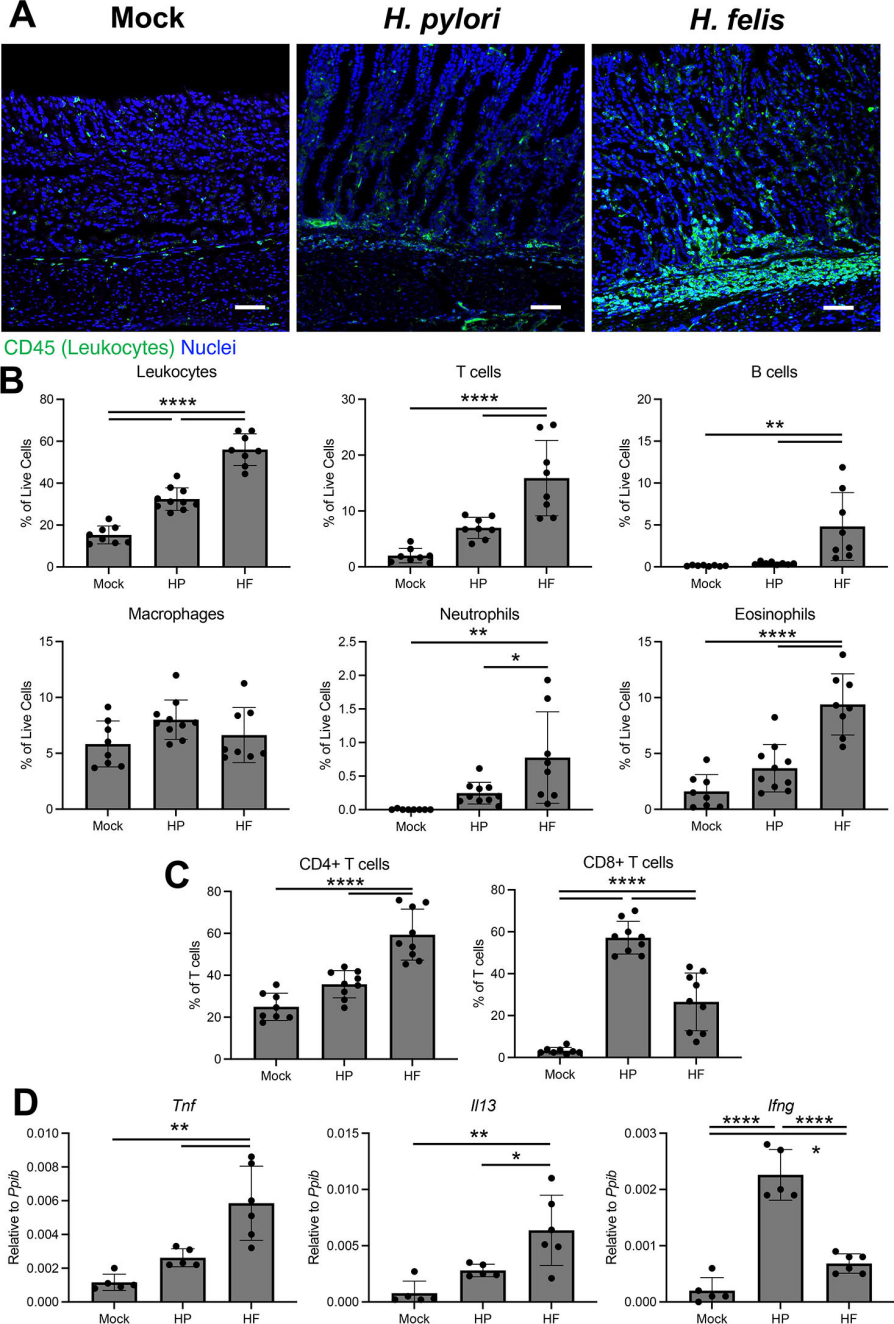
*H. felis-*infected mice develop more extensive gastric inflammation than *H. pylori*. Cryosections (**A**), cells (**B, C**), and RNA (**D**) were collected from the gastric corpus 2 months after mock infection or challenge with *H. pylori* or *H. felis*. (**A**) Representative immunostaining. Sections were probed with antibodies against the common leukocyte antigen CD45 (green). Nuclei were stained with DAPI. *n* ≥ 6. Scale bars = 100 µm. (**B, C**) Flow cytometry of the indicated cell types from disassociated gastric corpus. (**C**) CD4+ and CD8+ T cell ratios. *n* ≥ 8. (**D**) Quantitative RT-PCR of RNA from the gastric corpus for the indicated genes. *n* ≥ 5. *P ≤ 0.05, **P ≤ 0.01, ***P ≤ 0.001, ****P ≤ 0.0001.

Chronic inflammation associated with *Helicobacter* infection is a primary risk factor for gastric cancer initiation. Our studies demonstrate that HP and HF elicit robust macrophage activation *in vitro*. Next, we assessed the gastric immune-cell landscape 2 months post-challenge to determine how these bacterial species affect gastric inflammation. Immunostaining of the gastric corpus for the common leukocyte antigen CD45 revealed relatively few immune cells in the mock-challenged stomach, which is consistent with previous reports that the healthy stomach harbors relatively few resident leukocytes (Fig. 2A) (7, 32). HP infection induced modest leukocyte infiltration within the gastric corpus. In contrast, HF infection induced severe leukocyte infiltration throughout the entire gastric corpus (Fig. 2A). Next, we utilized spectral flow cytometry to investigate the specific immune-cell populations that respond to *Helicobacter* infection. For these studies, we disassociated the entire gastric corpus. Compared to mock controls, both HP and HF infection induced a statistically significant increase in gastric immune infiltration (Fig. 2B). However, the HF-infected stomach had significantly more inflammation than the HP-infected stomach. Both CD3+ T cells and B220+ B cells were significantly increased in HF-infected mice, with T cells being the most abundant immune-cell population (Fig. 2B). Although T cells were increased in HP-infected stomachs, the increfase was not statistically significant. Macrophages were not significantly changed by either *Helicobacter* infection. Neutrophils and eosinophils were only significantly increased in HF-infected mice.

Gastric-infiltrating T cells are linked to the bulk of the gastric atrophy associated with *Helicobacter* infection in mice (12). Gastric T cells are rare in the murine stomach during steady-state conditions (Fig. 2B), but those present in mock-infected mice were almost entirely CD4+ Helper T cells. Interestingly, although HP induced only a modest increase in total gastric T cells, each bacterial strain differentially affected the CD4/CD8 T cell ratios. HP induced a CD8+ biased T cell response, while HF induced a CD4+ biased T cell response (Fig. 2C). Finally, we assessed the expression of inflammatory genes within the gastric corpus by qRT-PCR. *Tnf* and *Il13* transcript levels were not significantly increased in response to HP infection, while both cytokines were significantly increased by HF infection (Fig. 2D). In contrast, *Infg* levels were significantly increased in both the HP- and HF-infected groups but were highest in HP-infected mice. Together, these results demonstrate that although the HP PMSS1 strain induces gastric inflammation, it is modest when compared to HF.

### *H. felis* induces more advanced atrophic gastritis than *H. pylori* in mice

**Fig 3:**
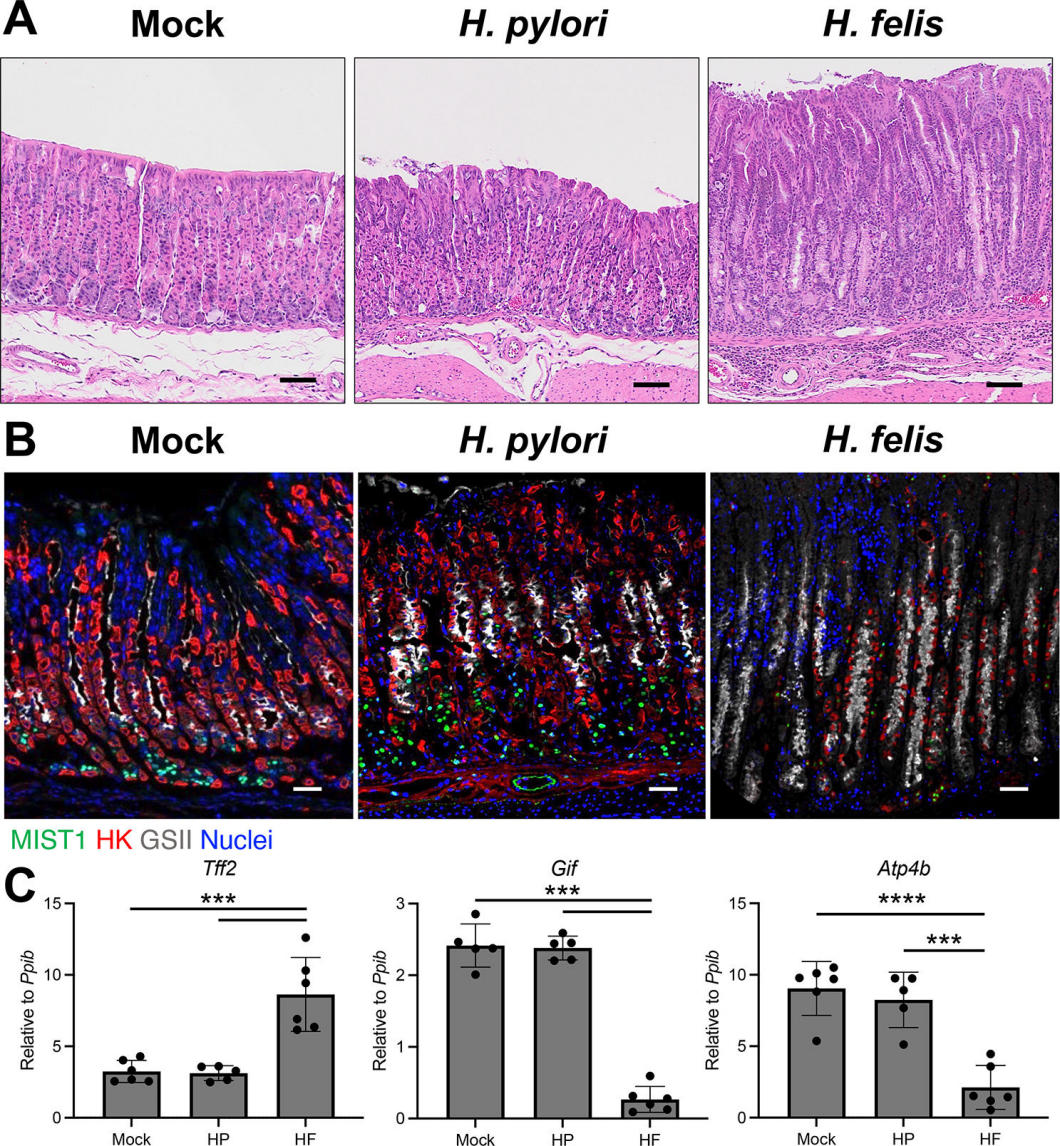
*H. felis-*infected mice develop more extensive atrophic gastritis than *H. pylori*. Tissues (**A, B**) and RNA (**C**) were collected from the gastric corpus 2 months after mock infection or challenge with *H. pylori* or *H. felis*. (**A**) Representative H&E micrographs. (**B**) Representative immunostaining. Sections were probed with antibodies against the MIST1 (mature chief cells, green), the H+/K+ ATPase (parietal cells, red), and the *Griffonia simplicifolia* lectin (mucous neck cells, gray). Nuclei were stained with DAPI. (**A, B**) *n* ≥ 6. Scale bars = 100 µM. (**C**) Quantitative RT-PCR of RNA from the gastric corpus for the indicated genes. *n* ≥ 5. **P ≤ 0.01, ****P ≤ 0.0001.

Because HF induced significantly more overall immune-cell infiltration and gastric T cell recruitment, we hypothesized that gastric epithelial damage would also be more severe in HF-infected mice. The morphology of the gastric corpus was assessed by H&E. Compared to mock-infected controls, HP-infected mice exhibited a slight increase in inflammatory infiltrate, similar to our findings above in Figure 2. In addition, although the overall gland length appeared unchanged, there was a noticeable foveolar hyperplasia, with the pit cells extending down to the gland neck (Fig. 3A). However, the parietal cells and chief cell compartment appeared grossly normal. In stark contrast, HF induced significant changes to the gastric epithelium throughout the entire corpus with a massive increase of immune cells and thickening of the gastric mucosa (Fig. 3A). Moreover, there was a dramatic expansion of the gastric pit cells and mucous neck cells, as well as a total absence of parietal and chief cells. Next, we immunostained with the mucous neck cell marker GSII lectin, the mature chief cell marker MIST1 (BHLHA15), and the parietal cell-specific hydrogen–potassium ATPase to better visualize changes to the major gastric cell lineages. HP-infected mice appeared similar to mock-infected controls, with normal proportions of mucous neck, chief, and parietal cells (Fig. 3B). Moreover, qRT-PCR for the cell lineage markers *Tff2* (mucous neck cells)*, Gif* (chief cells), and *Atp4b* (parietal cells) demonstrated that the relative expression of these major gastric lineages did not change 2 months post-HP challenge (Fig. 3C). In contrast, HP-infected mice exhibited dramatically increased GSII immunostaining, complete absence of MIST1+ cells, and only a few scattered parietal cells (Fig. 3B). Similarly, qRT-PCR demonstrated a significant expansion of *Tff2* and a significant 8.9-fold and 4.3-fold loss of *Gif* and *Atp4b,* respectively (Fig. 3C). These results demonstrate that HP infection induces non-atrophic gastritis in the gastric corpus within 2 months post-challenge, but that HF quickly induces atrophic gastritis denoted by massive damage to the gastric epithelium, leading to dramatic changes to the major gastric epithelial cell lineages.

### *H. felis* infection induces pyloric metaplasia

**Fig 4:**
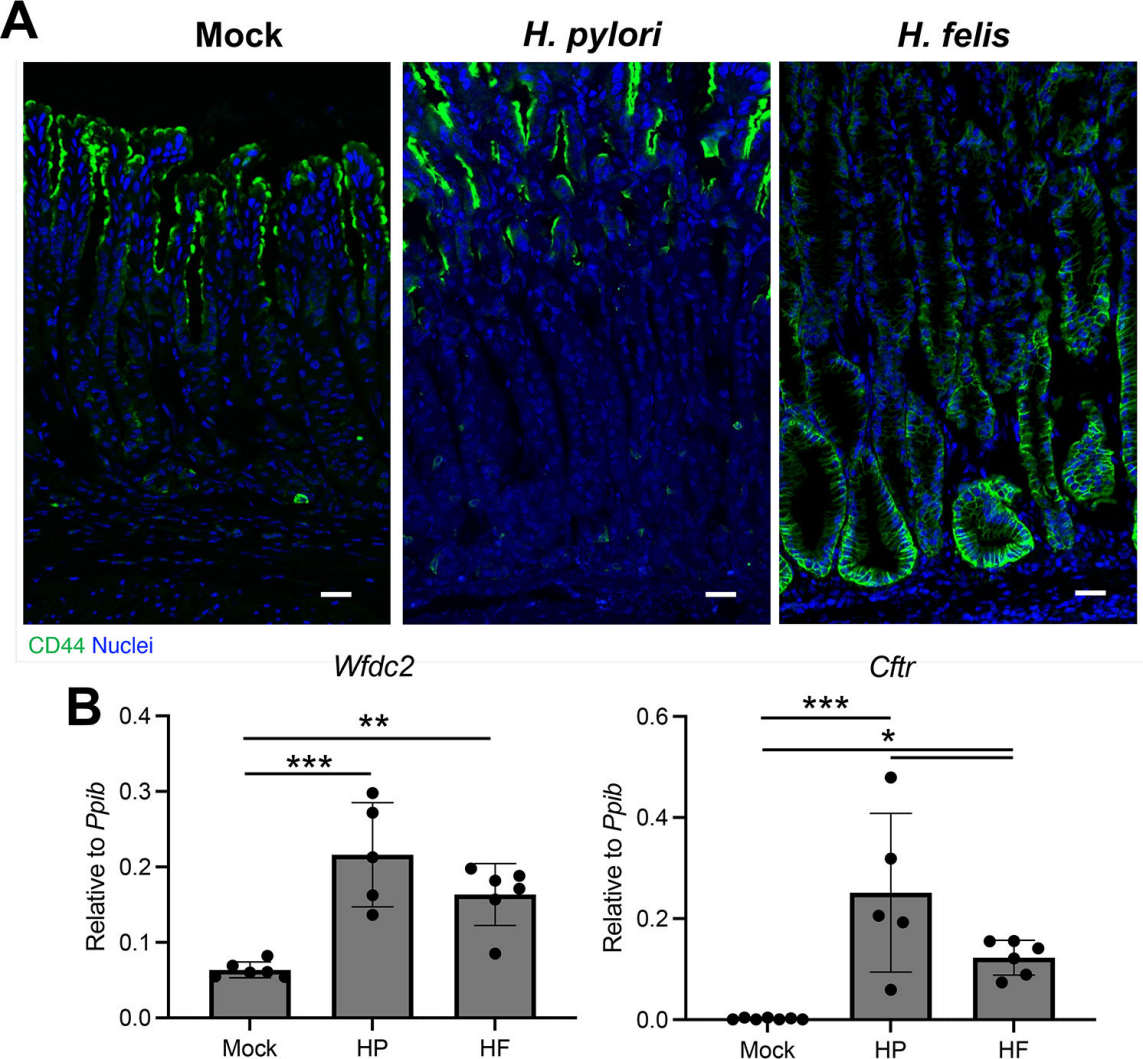
Pyloric metaplasia is more extensive in *H. felis*-infected mice. Tissues (**A**) and RNA (**B**) were collected from the gastric corpus 2 months after mock infection or challenge with *H. pylori* or *H. felis*. (**A**) Immunostaining for the pyloric metaplasia marker CD44v9. *n* ≥ 5. Scale bar = 100 µM. (**B**) Quantitative RT-PCR of RNA from the gastric corpus for the indicated genes. *n* ≥ 6. **P ≤ 0.01, ****P ≤ 0.0001.

The gastric corpus responds to inflammation and glandular damage by modifying cellular differentiation, leading to the development of pyloric metaplasia (PM), a putative preneoplastic lesion (33). To assess PM development, we immunostained with the *de novo* PM marker CD44v9 (34). Mock-infected mice did not exhibit any detectable CD44v9 expression within the glands of the gastric corpus (Fig. 4A). Similarly, CD44v9 staining was largely absent within the corpus glands of HP-infected mice, although there were occasionally positive cells within the gland base. In contrast, HF-infected mice exhibited widespread CD44v9-positive glands throughout the gastric corpus glands. Next, we assessed the expression of the PM marker transcripts *Wfdc2* and *Cftr*. Curiously, these transcripts were significantly increased in both HP- and HF-infected mice (Fig. 4B), and *Cftr* expression was significantly higher in HP-infected mice compared to HF-infected mice. Overall, HP-infected mice do not have gross morphological features of PM (Fig. 4A and B) and lack expression of CD44v9 (Fig. 4A) at 2 months post-infection.

### *H. felis* drives more extensive inflammation in the gastric antrum

**Fig 5:**
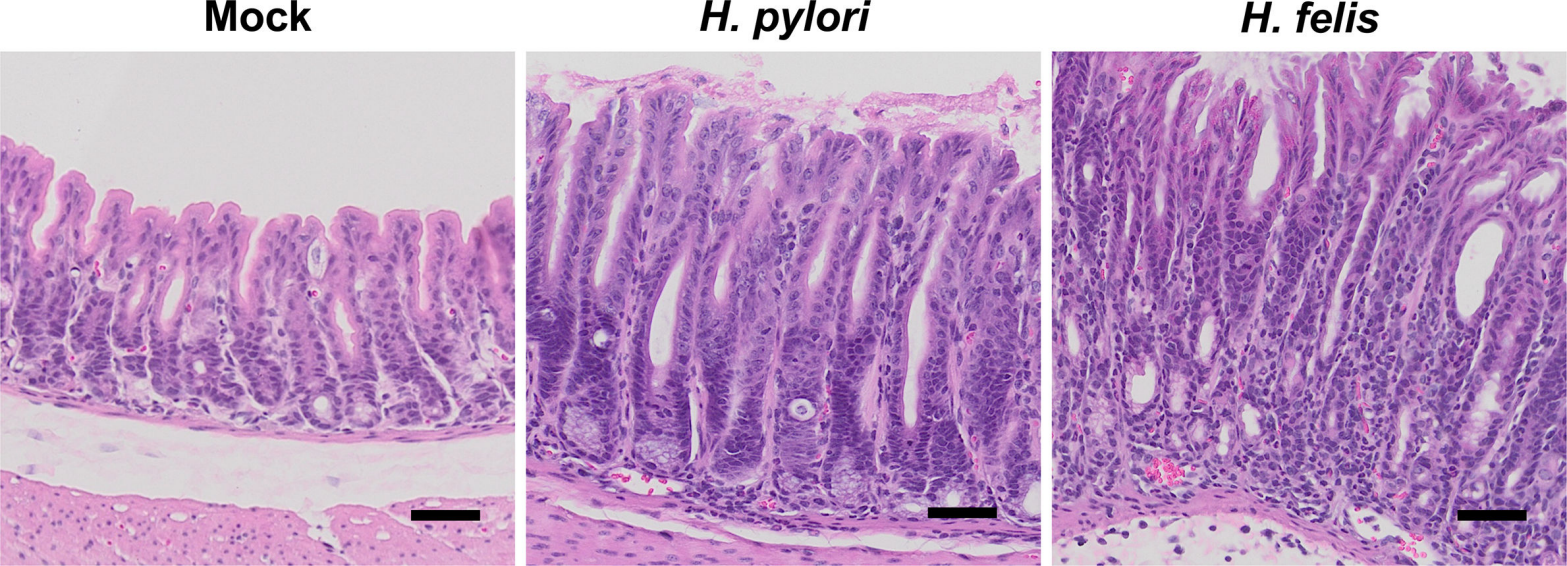
*H. felis* induces more extensive inflammation within the gastric antrum. H&E micrographs of the gastric antrum collected 2 months after mock infection or infection with *H. pylori* or *H. felis*. Scale bar = 50 µM. *N* ≥ 5.

In humans, HP typically initially colonizes the gastric antrum and gradually follows a front of inflammation and atrophy into the gastric corpus (33). PMSS1 follows a similar colonization pattern, although colonization patterns are strain specific (35). Next, we used H&E micrographs to assess the gross morphology of the gastric antrum in HP- and HF-infected mice. Compared to mock-infected controls, the antrum of HP- and HF-infected mice developed modest leukocyte infiltration (Fig. 5). However, leukocyte infiltration was more extensive in HF-infected mice, and the glands were thickened. These results indicate that HF drives more extensive inflammation throughout the entire stomach, affecting both the corpus and pylorus.

## DISCUSSION

Gastric-colonizing *Helicobacter* species have a complex relationship with the host immune response. Mild inflammation likely benefits the pathogen by causing oxyntic atrophy, allowing further colonization of the gastric corpus (33, 36) and increasing the expression of adhesion molecules by damaged and metaplastic gastric glands (37, 38). Severe inflammation is linked to lower bacterial loads due to increased pathogen clearance by the immune system and increased competition due to stomach colonization by intestinal flora as a result of gastric achlorhydria (39, 40). Severe inflammation ultimately may lead to *Helicobacter* eradication by the host. Therefore, HP has evolved to evade the host immune system (30). HP LPS lipid A is tetra-acetylated and poorly activates TLR4 (28, 41). HP flagella mutations at the TLR5 binding site evade TLR5 activation (31), and HP DNA is modified to avoid TLR9 activation (42). Rather, HP and HF are potent activators of TLR2 (28, 43). HF LPS has a similar effect on TLR4 activation, but unlike HP, it is reported to activate TLR9 to induce gastric inflammation (44). Our RNAseq analysis revealed that HF expressed higher levels of LPS synthesis genes and flagellar genes than HP. Moreover, HF induced significantly more robust inflammatory responses in mice. We, therefore, hypothesized that HF is innately more immunogenic than HP. Surprisingly, although HF induced higher mRNA levels of Il6 and Il1b, both bacterial species induced similar cytokine responses when assessed by a semi-quantitative dot plot array. Similar findings were reported by Mandell et al., where both HP SS1 and HF elicited similar activation of TLR4 and TRL2 (28). These data suggest that the enhanced inflammation observed in HF-colonized mouse stomachs is not due to enhanced immunogenicity of HF and may result from other mechanisms such as enhanced HF colonization or HP-mediated suppression of inflammatory pathways.

Our mouse colonization studies demonstrated that HF induced significantly more extensive gastric inflammation and epithelial remodeling than the HP PMSS1 strain. Chronic inflammation is necessary and sufficient for gastric cancer initiation. Mice that lack mature B and T cells are protected from morphological changes to the gastric epithelium in response to infection by either HP or HF (12, 45), and myeloid cell activation is required for pyloric metaplasia initiation (7, 8, 32). Moreover, overexpression of the proinflammatory IL1B or IFN-gamma under control of the parietal cell-specific H+/K+ ATPase β promoter drives gastric neoplasia development without accompanying *Helicobacter* infection (5, 6). Although we found that both HP and HF induced similar macrophage activation *in vitro*, gastric inflammation 2 months post-infection was significantly different for these two bacterial species. HP elicited only mild inflammatory responses within the gastric corpus 2 months post-infection. In contrast, HF induced severe inflammation throughout the entire gastric corpus at the same time point. Although previous studies reported that HP triggered gastric inflammation, atrophy, and metaplasia by 2 months post-infection (20, 46, 47), our findings indicate that these events are mild and restricted to the gastric corpus lesser curvature. In humans, HP typically initially colonizes the gastric antrum and, over time, spreads through the corpus lesser curvature (48). Although the underlying mechanisms of this pattern of spread through the stomach are not fully defined, the lesser curvature is more susceptible to inflammation and consequential oxyntic atrophy (8, 33). In contrast, HF-induced metaplasia and inflammation were ubiquitous throughout the gastric corpus. Within the gastric antrum, both bacterial species induced inflammation but as within the corpus, HF triggered more extensive inflammation. Poor colonization by the PMSS1 strain may account for some of the poor inflammation. Sigal et al. found that HP PMSS1 is present in the antrum but does not colonize the gastric corpus glands of C57BL/6J mice 2 months post-challenge (49). Moreover, interactions with the microbiota may abrogate HP-induced inflammation as the same study reported that C57BL/6 mice from different vendors exhibit dramatic differences in corpus inflammation (49). It is important to note that HP colonization patterns vary significantly by strain. Other HP strains, such as X47, preferentially colonize the corpus, although this strain does not express a CagA (35).

T cells are required for the bulk of gastric epithelial damage and remodeling associated with *Helicobacter* infection in mice (12, 50). Elevated levels of Th1/Th17-associated cytokines are linked to increased cancer risk in humans (51, 52). Here, we found that HP does not induce a significant T cell response 2 months post-infection. This lack of response is consistent with the mild epithelial changes and lack of metaplasia development. HF induced dramatic T cell activation, particularly in the CD4+ T cell compartment. CD4+ T cells are reported to drive the bulk of gastric damage associated with HF infection (53). Interestingly, although HP only caused mild gastric T cell recruitment, it caused a shift in the CD4/CD8 T cell ratio, skewing toward an enhanced CD8 cytotoxic T cell response. This increase is likely due to the CagA pathogenicity island and functional T4SS that transfers bacterial components into host epithelial cells (54). We found that although HF inflammation was predominantly biased toward CD4+ T cells, there still was an overall increase in gastric CD8+ T cells. CD8 T cells have also been reported to induce gastric damage during HF infection, but their role is secondary to CD4 T cells (55). The exact role of these cytotoxic T cells is unclear. Although CD8+ T cell responses contribute to epithelial damage (54, 55), they undoubtedly also play important roles in responding to neoantigens to remove developing tumor cells. The hyper-inflamed environment induced by *Helicobacter* may promote cytotoxic T cell exhaustion and dysfunction, potentially explaining why more intense gastric inflammation is linked to increased cancer risk.

HP and HF are closely related *Helicobacter* species, sharing more than 60% of each other’s genome (23). HF’s genome encodes many HP orthologs that are required for gastric colonization, such as a urease cluster, flagellar and chemotactic genes, gastric epithelial adhesion proteins, RecA, collagenase, and iron uptake gene clusters. CagA and the vacuolating toxin VacA are the most well-known HP virulence factors associated with gastric cancer risk in humans and are notably absent from the HF genome (23). CagA is a bona fide bacterial oncoprotein that is injected into host epithelial cells through its T4SS (56). Once inside host cells, CagA attaches to the plasma membrane and promiscuously interacts with host proteins, inducing mutations, cellular reprogramming, and inflammation (57). Transgenic mice that ectopically express CagA from parietal cell-specific H+/K+ ATPase promoter spontaneously develop gastric tumors (58). Although CagA is sufficient to drive gastric inflammation and cancer initiation, our results indicate that inflammation induced by CagA+ HP still pales in comparison to HF. CagA expression is likely one of the most important rationales for using HP in mice infection studies. However, when HP was re-isolated from infected mice, it was found that they had lost their T4SS functionality by 3–4 months post-infection (20, 21). Thus, the selection of HP for mouse infection studies strains solely on their expression of CagA may not yield the desired phenotypes. Although we showed that HF induced more robust inflammation and epithelial remodeling than HP, it should be noted that we did not assess changes within gastric epithelial cells. CagA has multiple effects on epithelial cells, affecting cell signaling pathways, suppressing p53 and other tumor suppressors, and altering cellular proliferation (59–61). Thus, study goals and experimental endpoints should be carefully considered to select the appropriate *Helicobacter* model.

In summary, HP infection studies in mice mount specific challenges in modeling the inflammatory phenotypes and cancer initiation observed in humans. These problems stem from the fact that HP is not a natural mouse pathogen and common mouse-adapted strains such as PMSS1 suffer from weak immunogenicity and functional loss of virulence factors that promote cancer risk. HF has been used for decades to model pro-neoplastic gastric inflammation and cancer initiation, and quickly and consistently initiates inflammation in mouse models. Although HF is more strongly suited for studies on *Helicobacter-*induced gastric inflammation, metaplasia, and cancer initiation, its lack of CagA and VacA limits its utility for studies on the direct effects of the bacteria on gastric epithelial cells. Moreover, HP is likely better suited for studies on bacterial colonization and interactions with the gastric microbiota.
